# Evaluation of the Impact of Micellar Formulation of Resveratrol on Neurotrophic Factors and Neuromodulatory Potential in Healthy Rats

**DOI:** 10.3390/molecules31142536

**Published:** 2026-07-21

**Authors:** Maria Lazarova, Miroslava Stefanova, Elina Tsvetanova, Almira Georgieva, Krasimira Tasheva, Lyubomira Radeva, Krassimira Yoncheva

**Affiliations:** 1 Department of Synaptic Signaling and Communication, Institute of Neurobiology, Bulgarian Academy of Sciences, Acad. G. Bonchev Str., 23, 1113 Sofia, Bulgaria; 2 Radioisotope Laboratory, Institute of Neurobiology, Bulgarian Academy of Sciences, Acad. G. Bonchev Str., 23, 1113 Sofia, Bulgaria; mira_stefanova@mail.bg; 3 Department of Biological Effects of Natural and Synthetic Substances, Institute of Neurobiology, Bulgarian Academy of Sciences, Acad. G. Bonchev Str., 23, 1113 Sofia, Bulgaria; elina_nesta@abv.bg (E.T.); almirageorgieva@gmail.com (A.G.); 4 Department of Plant Ecophysiology, Institute of Plant Physiology and Genetics, Bulgarian Academy of Sciences, Acad. G. Bonchev Str., 21, 1113 Sofia, Bulgaria; krasitasheva@abv.bg; 5 Faculty of Pharmacy, Medical University of Sofia, 1000 Sofia, Bulgaria; l.radeva@pharmfac.mu-sofia.bg

**Keywords:** resveratrol, Pluronic micelles, neuromodulation, acetylcholinesterase, BDNF, pCREB, monoamines, oxidative stress, healthy rats

## Abstract

Encapsulation of resveratrol (RVT), a natural polyphenol, in mixed Pluronic F127/P123 micelles (mRVT) has enhanced its pharmacological efficacy in experimental dementia models. The aim of this study was to evaluate neurochemical and behavioral parameters of mRVT treatment in healthy subjects. For this purpose, pure RVT and mRVT (both at 10 mg/kg i.p.) were applied for 9 consecutive days in healthy male Wistar rats. Memory performance was assessed using the novel object recognition and passive avoidance tests. Neurotransmitter levels, neurotrophic and transcription factors, and oxidative stress markers were quantified in the cortex and hippocampus using enzyme-linked immunosorbent assay kits. Our results showed that neither treatment altered locomotor activity or short-term memory performance. Both RVT and mRVT treatments reduced hippocampal acetylcholinesterase activity to a similar extent and elevated acetylcholine levels in the cortex and hippocampus, with mRVT producing significantly more pronounced effect in the hippocampus. Compared to pure RVT, mRVT elicited a greater increase in noradrenaline and serotonin levels in both brain regions and comparably upregulated BDNF and pCREB protein levels in the cortex. Furthermore, mRVT uniquely reduced cortical lipid peroxidation—an effect absent with pure RVT treatment. These findings demonstrate that micellar encapsulation substantially amplifies the neurochemical efficacy of resveratrol under physiological conditions without adverse behavioral effects.

## 1. Introduction

Alzheimer’s disease (AD) is a prominent age-related progressive neurodegenerative disorder characterized by a gradual decline in cognitive functions [1]. AD patients typically experience deficits in working, spatial, and anterograde memory [2]. The progression of the disease triggers cognitive deterioration through multiple interrelated mechanisms: compromise of the brain’s cholinergic framework, because of a substantial loss of cholinergic neurons within the basal forebrain; diminished levels of the neurotransmitter acetylcholine (ACh) and elevated acetylcholinesterase (AChE) activity [3,4]; dysregulation of the monoaminergic system, characterized by extensive neuronal loss and reduced levels of neurotransmitters, receptors, and metabolites within the dopaminergic (DA), noradrenergic (NA), and serotonergic (Sero) systems [5]; and an increase in cerebral oxidative stress levels, which directly contributes to memory impairment by disrupting synaptic plasticity and promoting neuronal loss [6,7]. In addition, the elevated levels of reactive oxygen species (ROS) have been shown to downregulate the brain-derived neurotrophic factor (BDNF)/cAMP-response element-binding protein (pCREB) signaling pathway, further compromising hippocampal-dependent memory encoding [8,9,10]. In this context, compounds with multimodal mechanisms that simultaneously modulate several neurochemical pathways represent a promising therapeutic strategy for the treatment of neurodegenerative diseases.

Resveratrol (3,4′,5-trihydroxystilbene, RVT) is a natural polyphenol present in dietary sources, such as grapes, berries, and wine. It has recently attracted great interest due to its potentially beneficial effects on human health, including cardioprotective, anti-inflammatory, anticancer, antidiabetic, and neuroprotective effects [11]. Moreover, RVT has been reported to improve both cognitive and non-cognitive function, such as learning, memory, anxiety, and depression, and has shown promising results in AD models [12,13]. The potential molecular mechanisms underlying the neuroprotective properties of resveratrol include: modulation of the expression and activity of SIRT1, a NAD-dependent protein deacetylase, which promotes neurite outgrowth and enhances neural plasticity in the hippocampal region [14]; activation of AMP-activated protein kinase (AMPK), known as a stimulator of neuronal differentiation and mitochondrial biogenesis [15]; antioxidant activity by decreasing levels of malondialdehyde in the cerebral cortex and hippocampus, while also increasing superoxide dismutase activity and glutathione levels [16]; a BDNF-preserving effect [17], a pivotal molecule for hippocampal plasticity [18]; and a stimulatory effect on brain monoamine levels [19] and significant anti-inflammatory activity [20].

The tolerability and safety profile of resveratrol is very high, and no clinically significant pharmacological interaction of this nutraceutical with conventional drugs is known. Despite its therapeutic potential, the clinical utility of RVT is severely limited by poor oral bioavailability and a short serum half-life [21]. To address these challenges, recent pharmaceutical developments have focused on advanced delivery systems designed to overcome these pharmacokinetic limitations [22,23]. In our previous research, mixed Pluronic micelles were utilized as a nanocarrier for resveratrol [24,25,26]. The in vivo findings revealed that the micellar resveratrol (mRVT) exerted higher pharmacological activity than the pure drug in rats with experimental dementia. This was evidenced by stronger AChE-inhibitory activity, increased noradrenaline neurotransmission, and activation of the BDNF/CREB signaling pathway. Moreover, pronounced anti-apoptotic effects were observed in the cortex and hippocampus of the mRVT treated animals. These factors contribute to superior neuroprotective properties and improved learning and memory. The dose of 10 mg/kg was identified as the most effective [24]. Additionally, in vitro and in vivo studies highlighted the enhanced antioxidant capacity and protective activity of the micellar resveratrol against nuclear DNA damage in in vitro models of oxidative stress [25,26]. The data indicated that the micellar formulation is a promising platform for therapeutic interventions against neurodegenerative processes.

Despite these encouraging findings under experimental models of cognitive impairment, the neurochemical and behavioral parameters of the micellar resveratrol under physiological conditions remain insufficiently explored. Such investigations are essential for distinguishing between true therapeutic effects and nonspecific alterations in normal brain function. Therefore, the aim of the present study was to evaluate the effects of pure and micellar resveratrol on locomotor activity, memory performance, and key neurochemical parameters, including cholinergic function, monoamine levels, and oxidative status, in the cortex and hippocampus of healthy rats.

## 2. Results

### 2.1. The Incorporation of Resveratrol in Polymeric Micelles

The incorporation of hydrophobic active substances in polymeric micelles is considered an appropriate strategy to boost their bioavailability and application by increasing their aqueous solubility. Indeed, the incorporation of resveratrol in the micelles resulted in the preparation of aqueous nanodispersion with concentration of resveratrol reaching 1210 µg/mL. This is a significant increase in resveratrol solubility bearing in mind that its solubility in water is 50 µg/mL. Moreover, the small size of these nanostructures can significantly enhance the intracellular transport. The resveratrol-loaded micelles prepared in our study possessed a mean diameter of 33 ± 2 nm and a narrow size distribution (PDI of 0.278), which can enhance the direct cell penetration via passive diffusion. The zeta potential was slightly negative (−4.0 ± 0.06 mV). Despite the close to zero zeta potential, the hydrophilic shell (comprised from PEO) of the micelles allows high colloidal stability owing to a steric stabilization. However, this could also have impact on the possible side effects of the substance. Therefore, we evaluated the safety profile of the resveratrol-loaded micelles.

### 2.2. mRVT Treatment Does Not Alter Locomotor Activity

**Figure 1:**
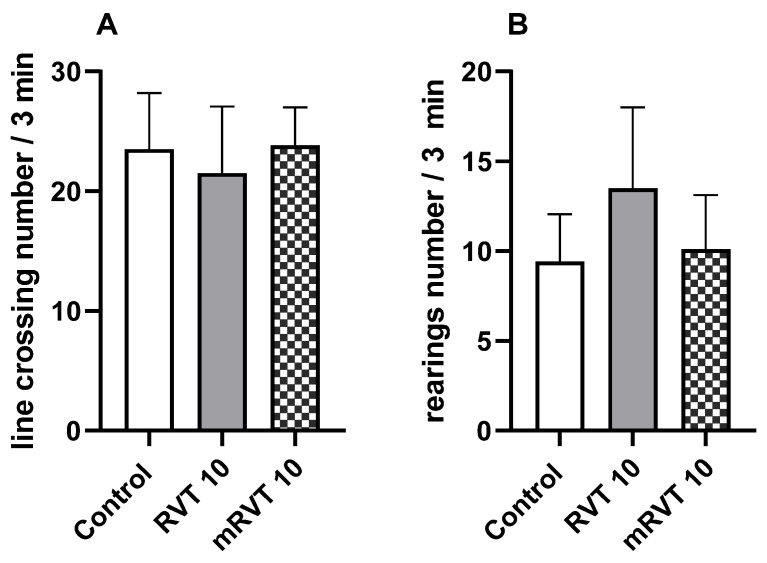
Effects of RVT and mRVT on locomotor activity in healthy animals. (**A**) Number of line crossings and (**B**) number of rearings during a 3 min test period. Both compounds were administered at a dose of 10 mg/kg. Data are expressed as mean ± SEM (*n* = 9 per group). Statistical analysis was performed using one-way ANOVA.

Our results demonstrated that RVT and mRVT, both at a dose of 10 mg/kg, did not significantly alter locomotor activity under physiological conditions. No significant changes were observed in the number of line crossings or rearings during the 3 min test period (Figure 1A,B).

### 2.3. mRVT Treatment Does Not Affect Short-Term Memory

**Figure 2:**
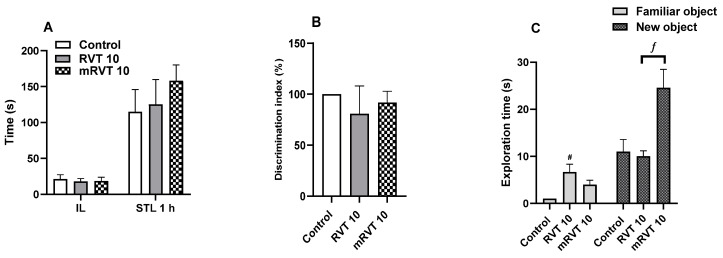
Effects of RVT and mRVT on memory performance in healthy animals. (**A**) Step-through latency in the passive avoidance test; (**B**) discrimination index; and (**C**) exploration time in the novel object recognition test. Both compounds were administered at a dose of 10 mg/kg. Data are expressed as mean ± SEM (*n* = 9 for control, *n* = 7 for RVT, and *n* = 7 for mRVT groups). Statistical analysis was performed using one-way ANOVA, followed by Tukey’s post hoc test. ^#^ *p* < 0.05 vs. control; ^ƒ^ *p* < 0.05 RVT vs. mRVT.

As shown in Figure 2A,B, the administration of RVT and mRVT did not significantly alter memory performance in either the passive avoidance or the novel object recognition (NOR) tests. The effect of treatment in the NOR test was evaluated via calculation of discrimination index (DI) as a primary outcome measure and individual exploration times for each object as a secondary measure. According to our results no significant differences in the DI were observed between the groups (Figure 2B). However, the time spent exploring the novel object differed significantly between the groups (F(2, 20) = 10.042, *p* = 0.001, η^2^ = 0.501). As illustrated in Figure 2C, the animals in the mRVT group spent significantly more time exploring the novel object (24 s) compared to the control and RVT groups (10 s) (*p* < 0.05) (Table 1).

### 2.4. mRVT Treatment Modulates Cholinergic Markers

**Figure 3:**
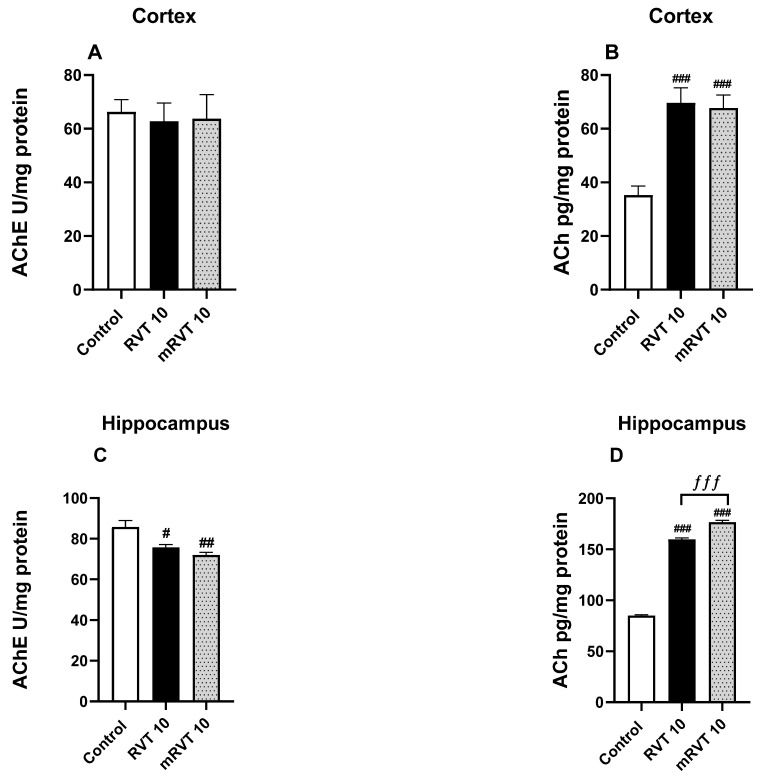
Effects of RVT and mRVT on AChE activity (**A**,**C**) and ACh content (**B**,**D**) in the cortex and hippocampus of healthy rats. Both compounds were administered at a dose of 10 mg/kg. Data are expressed as mean ± SEM (for AChE activity analysis, *n* = 6 for control, *n* = 5 for RVT, and *n* = 5 for mRVT groups; for ACh analysis, *n* = 6 per group). Statistical analysis was performed using one-way ANOVA, followed by Tukey’s post hoc test. ^#^ *p* < 0.05; ^##^ *p* < 0.01; ^###^ *p* < 0.001 vs. control; ^ƒƒƒ^ *p* < 0.001 RVT vs. mRVT.

Our results showed that in the cortex, the treatment with RVT and mRVT (both at 10 mg/kg) did not significantly alter the control AChE activity of healthy animals (Figure 3A). However, ACh levels in the cortex were significantly increased after RVT (by 98%, *p* < 0.001) and mRVT (by 92%, *p* < 0.001) treatment.

In the hippocampus, basal AChE activity was decreased by 12% (*p* < 0.05) and 16% (*p* < 0.01) after RVT and mRVT application, respectively (F(2, 14) = 11.02, *p* = 0.0013, η^2^ = 0.611) (Figure 3C). Furthermore, RVT increased hippocampal ACh levels by 88% (*p* < 0.001), while mRVT led to an increase of 108% (*p* < 0.001) (F(2, 15) = 1056, η^2^ = 0.993) (Figure 3D). Post hoc analysis revealed significant differences between the effects of RVT and mRVT on the ACh levels in the hippocampus. In particular, the effect of mRVT was significantly more pronounced than that of RVT (by 11%, *p* < 0.001, *n* = 6).

### 2.5. mRVT Treatment Increases Monoamine Levels

**Figure 4:**
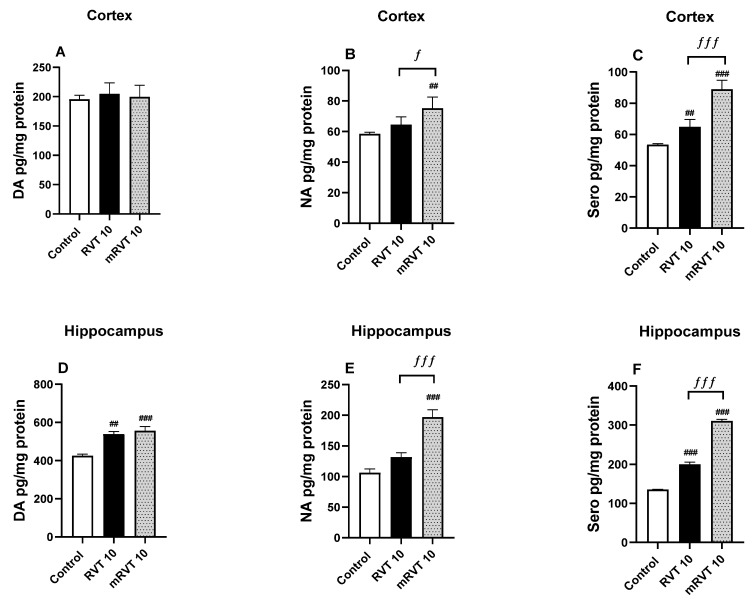
Effects of RVT and mRVT on DA, NA, and Sero content in the cortex (**A**–**C**) and hippocampus (**D**–**F**) of healthy rats. Both compounds were administered at a dose of 10 mg/kg. Data are expressed as mean ± SEM (*n* = 5 for control, *n* = 5 for RVT, *n* = 6 for mRVT groups). Statistical analysis was performed using one-way ANOVA, followed by Tukey’s post hoc test. ^##^ *p* < 0.01, ^###^ *p* < 0.001 vs. control; ^ƒ^ *p* < 0.05, ^ƒƒƒ^ *p* < 0.001 RVT vs. mRVT.

Our results revealed that cortical NA levels increased by 29% (*p* < 0.01) after mRVT administration but remained unchanged following RVT treatment (F(2, 13) = 13.33, *p* = 0.0007, η^2^ = 0.672), compared to the control group. Sero levels were similarly elevated, increasing by 21% (*p* < 0.01) after RVT and by 66% (*p* < 0.001) after mRVT treatment (F(2, 13) = 91.32, *p* < 0.0001, η^2^ = 0.934) (Figure 4B,C).

Furthermore, post hoc analysis indicated significant differences between the effects of RVT and mRVT within the cortex, demonstrating that mRVT produced a significantly greater increase in NA levels (by 17%, *p* < 0.05, *n* = 6) and Sero levels (by 37%, *p* < 0.001, *n* = 6) compared to RVT, as depicted in Figure 4C.

Regarding the hippocampus, the application of RVT resulted in an increase in the control levels of DA (by 26%, *p* < 0.01) and Sero (by 48%, *p* < 0.001), whereas NA content was not significantly changed (Figure 4D–F). The administration of mRVT resulted in a more substantial increase in DA levels (by 32%, *p* < 0.001) (F(2, 13) = 17.39, *p* = 0.0002, η^2^ = 0.728), NA levels (by 85%, *p* < 0.001) (F(2, 13) = 25.14, *p* < 0.0001, η^2^ = 0.795), and Sero levels (by 130%, *p* < 0.001) F(2, 13) = 472.1, *p* < 0.0001, η^2^ = 0.986) compared to the control group.

Post hoc analysis indicated significant differences between the effects of RVT and mRVT within the hippocampus, demonstrating that mRVT produced a significantly greater increase in NA levels (by 49%, *p* < 0.001, *n* = 6) and Sero levels (by 56%, *p* < 0.001, *n* = 6) compared to RVT, as depicted in Figure 4E,F.

### 2.6. mRVT Treatment Modulates BDNF and pCREB Protein Levels in the Cortex

**Figure 5:**
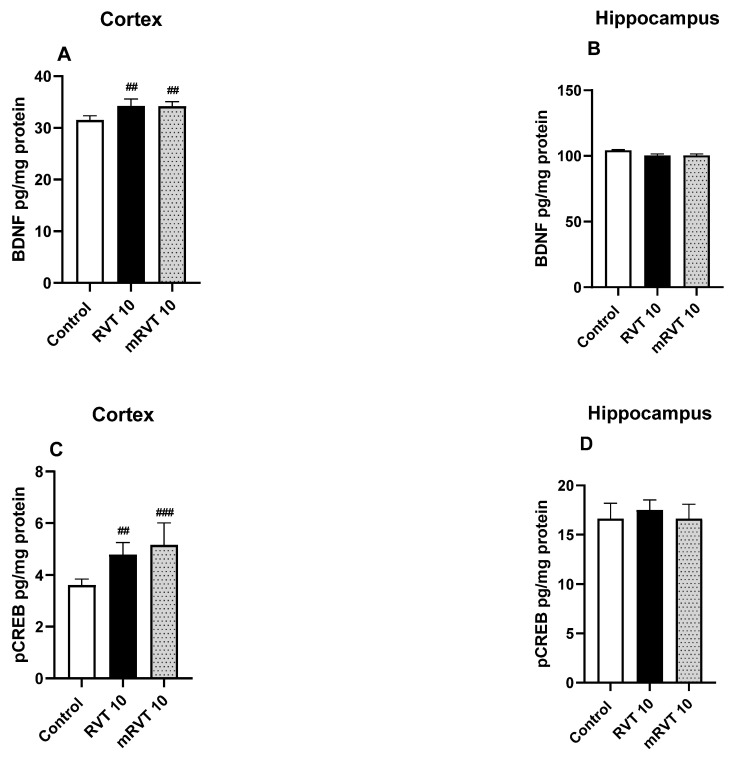
Effects of RVT and mRVT on BDNF and pCREB protein levels in the cortex (**A**,**C**) and hippocampus (**B**,**D**) of healthy rats. Both compounds were administered at a dose of 10 mg/kg. Data are expressed as mean ± SEM (*n* = 6 per group). Statistical analysis was performed using one-way ANOVA, followed by Tukey’s post hoc test. ^##^ *p* < 0.05, ^###^ *p* < 0.001 vs. control.

As illustrated in Figure 5A,B, administration of RVT and mRVT to healthy animals significantly increased control levels of BDNF protein content in the cortex by 9% (*p* < 0.01) and 8% (*p* < 0.01), respectively (F(2, 15) = 8.382, *p* = 0.0036, η^2^ = 0.528). In the hippocampus, the protein levels were not significantly affected.

Regarding pCREB protein levels, statistically significant changes were observed exclusively in the cortex (Figure 5C). Both treatments led to a marked increase in protein content compared to control values: 32% (*p* < 0.01) following RVT administration and 43% (*p* < 0.001) following mRVT treatment (F(2, 15) = 11.68, *p* = 0.0009, η^2^ = 0.609). No significant alterations in pCREB levels were detected in the hippocampus among the experimental groups (Figure 5D).

### 2.7. mRVT Treatment Decreases LPO Levels

**Figure 6:**
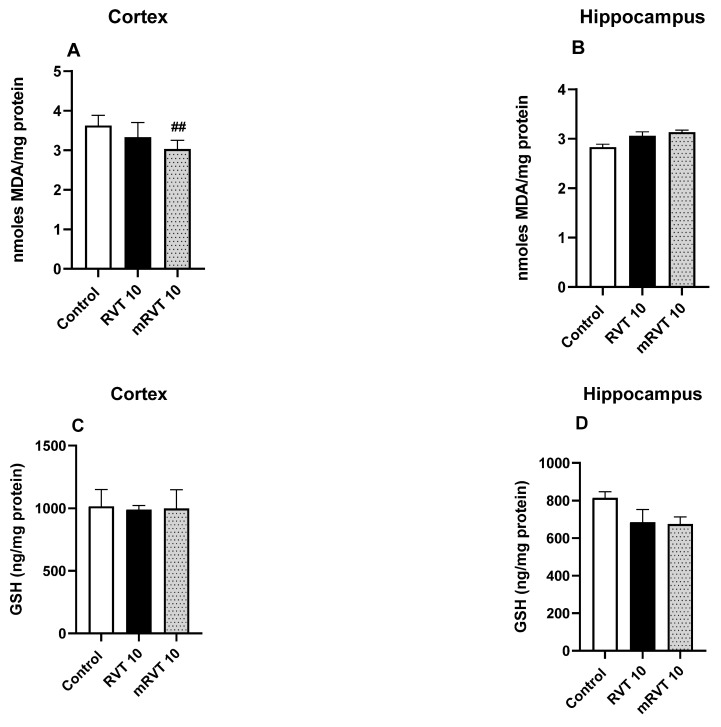
Effects of RVT and mRVT treatment on LPO and GSH levels in the cortex (**A**,**C**) and hippocampus (**B**,**D**) of healthy rats. Both compounds were administered at a dose of 10 mg/kg. Data are expressed as mean ± SEM (*n* = 6 per group). Statistical analysis was performed using one-way ANOVA, followed by Tukey’s post hoc test. ^##^ *p* < 0.01 vs. control.

As shown in Figure 6A, LPO levels were significantly decreased by 16% (*p* < 0.01) in the cortex after mRVT application (F(2, 15) = 7.637, *p* = 0.0052, η^2^ = 0.505). However, neither RVT nor mRVT treatment significantly altered LPO levels in the hippocampus. Similar observations were made regarding GSH levels in either the cortex or hippocampus (Figure 6B–D).

### 2.8. mRVT Treatment Increases Antioxidant Enzyme Activity

**Figure 7:**
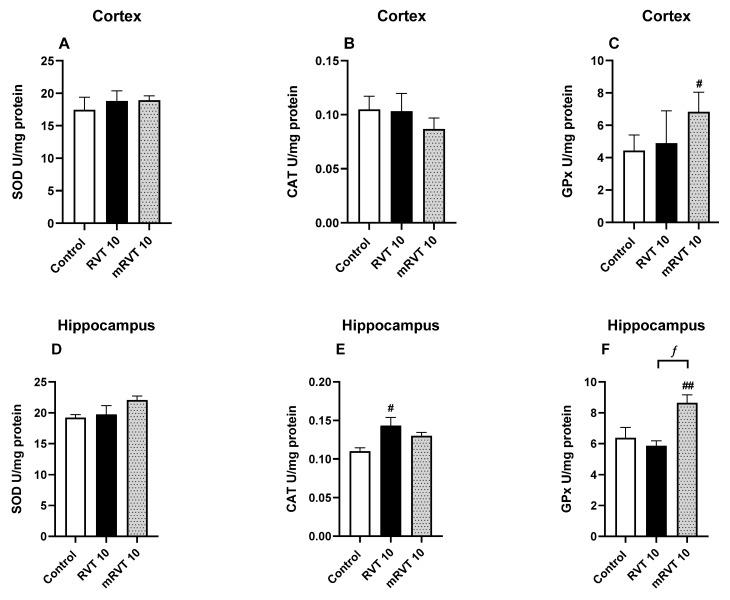
Effects of RVT and mRVT treatment on SOD, CAT, and GPx activity in the cortex (**A**–**C**) and hippocampus (**D**–**F**) of healthy rats. Both compounds were administered at a dose of 10 mg/kg. Data are expressed as mean ± SEM (*n* = 6 for control, *n* = 6 for RVT, and *n* = 5 for mRVT groups). Statistical analysis was performed using one-way ANOVA, followed by Tukey’s post hoc test. ^#^ *p* < 0.05; ^##^ *p* < 0.01 vs. control; ^ƒ^ *p* < 0.05 RVT vs. mRVT.

Our results demonstrated that CAT activity in the hippocampus was increased by 30% (*p* < 0.05) after RVT treatment compared to the control (F(2, 14) = 4.916, *p* = 0.0241, η^2^ = 0.413) (Figure 7E). Additionally, mRVT treatment significantly elevated GPx activity in both the cortex and hippocampus by 54% (F(2, 14) = 3.919, *p* = 0.0445, η^2^ = 0.359) and 36% (F(2, 14) = 7.287, *p* = 0.0068, η^2^ = 0.510), respectively. Post hoc analysis revealed significant differences between the effects of RVT and mRVT on hippocampal GPx activity, with mRVT exhibiting a markedly stronger effect (48% higher than RVT, *p* < 0.05, *n* = 6).

## 3. Discussion

The present study investigated the effects of resveratrol encapsulated in micelles (10 mg/kg) on behavioral and neurochemical parameters in healthy rats. This approach allowed us to identify the pharmacological activity of the encapsulated compound compared to the pure drug under physiological conditions, thereby complementing existing data regarding its efficacy under pathological conditions [24,25,26]. To ensure consistency with our previous work, we used the same behavioral tests to assess the behavioral performance of animals following all treatments: spontaneous locomotor activity and short-term recognition memory via NOR test. Additionally, we included the passive avoidance task to gain a more complete picture of short-term memory in healthy subjects.

NOR test was used to evaluate recognition memory in its short-term aspect based on the innate tendency of rodents to explore a novel object more extensively than a familiar one [27,28]. Recognition memory performance was assessed by calculating the discrimination index (DI) for all groups as the primary outcome measure and individual exploration times for each object as the secondary measure. DI represents the ratio of the time spent exploring the novel object to the total exploration time of both objects and, according to our results, did not differ significantly between the groups. The individual exploration times for each object, presented separately, show that animals in the mRVT group spent significantly more time exploring the novel object compared to both the control and RVT groups (*p* < 0.05), suggesting a trend toward improved object discrimination. Given the absence of a significant DI difference, firm conclusions regarding memory enhancement cannot be drawn. These findings align with our previous study, where we tested the effects of RVT and mRVT (5 and 10 mg/kg) on recognition memory in rats with experimental dementia. The data showed that the mRVT group (10 mg/kg) spent the most time exploring the novel object, maintaining a DI level similar to the control [26].

To further evaluate the short-term memory, the passive avoidance task was employed as a complementary behavioral assay. In this paradigm, animals learned to associate a specific environment with an aversive stimulus, such as a foot-shock. Rodents with intact memory function typically exhibited a tendency to refrain from entering the dark compartment where the stimulus was previously encountered [29,30]. Our findings revealed an increase in step-through latency within the mRVT-treated healthy rats compared to both the control and RVT groups; however, this did not reach statistical significance. These results indicate that both compounds do not exert nonspecific stimulatory or sedative effects. Moreover, neither RVT nor mRVT significantly altered the basal locomotor activity of healthy animals, suggesting that motor function remained unimpaired. Such observations are particularly important, as they suggest a lack of behavioral interference under physiological conditions.

The enhancement effects of resveratrol on recognition memory, spatial working memory, and motor coordination in healthy aged rats have been reported. However, these findings pertain to the non-pathological aging process and involve more prolonged treatment protocols [19,31,32].

In contrast to the absence of statistically significant behavioral changes, both RVT and mRVT significantly increased acetylcholine (ACh) levels in the cortex and hippocampus. They exhibited significant AChE inhibitory activity in the hippocampus. The AChE inhibitory activity of the resveratrol has been documented in the literature [33]. The effect of mRVT on enzyme activity was more pronounced than that of RVT. These findings are in line with previous reports by Lazarova et al., who demonstrated that micellar resveratrol treatments (10 mg/kg) in rats with induced cognitive impairment initiated a more potent AChE inhibitory activity in the hippocampus, whereas both formulations of RVT and mRVT (5 and 10 mg/kg) possessed an equal neuroprotective ACh-preserving effect [24].

It should be noted that, despite the fact that ELISA-based quantification of ACh and monoamines in tissue homogenates primarily captures intracellular and vesicular stores rather than active synaptic release, we observed a significant modulatory effect of the treatment on the markers examined.

There have been data reported establishing the relation between resveratrol’s positive impact on cognitive function and its ability to modulate brain monoaminergic neurotransmission in healthy subjects. Research by Sarubbo et al. demonstrated that chronic (40 weeks) resveratrol (20 mg/kg) administration in healthy aged rats (20 months) increases DA, NA, and Sero levels, as well as the activity of key enzymes involved in monoamine synthesis within critical brain structures [19]. Our results showed a modulatory effect of both compounds treatment on monoaminergic markers in healthy young rats (2 months), even with a shorter administration period (9 days). The mRVT produced a more pronounced effect than pure RVT, particularly on noradrenaline and serotonin brain levels, suggesting a possible modulatory role of micellar encapsulation in the neurochemical efficacy of resveratrol. These results are in line with our previous research, which demonstrated the superior noradrenaline-modulating effect of mRVT (10 mg/kg) under pathological conditions [24].

As a key modulator of synaptic plasticity, BDNF plays a vital role in cognition, learning, and memory formation, making it a critical molecule in the context of neurodegenerative diseases [34]. Rahvar et al. demonstrated for the first time that oral resveratrol treatment induces BDNF mRNA expression in the hippocampus of healthy rat brains [35]. A pivotal finding of our study is the differential modulatory effect of RVT and mRVT on the BDNF/pCREB signaling axis in healthy rats. Under basal physiological conditions, both RVT and mRVT treatment comparably upregulated BDNF and pCREB protein levels in the cortex. No significant changes were detected in the hippocampus for either marker. These results are consistent with our previous observations in pathological dementia models, where mRVT at 10 mg/kg was uniquely capable of significantly enhancing pCREB expression across both brain structures [24]. Taken together, these data suggest that micellar encapsulation effectively amplifies the bioavailability and intrinsic neurotrophic support provided by resveratrol, both with and without a pathological challenge.

The antioxidant properties of resveratrol, which underlie its neuroprotective effects in pathological models, are widely documented [36]. Administration of RVT in healthy rats decreases levels of malondialdehyde and stimulates activity of antioxidant enzymes, such as CAT and SOD [36]. In the present study, we evaluated five key oxidative stress markers, including both enzymatic (SOD, CAT, GPx) and non-enzymatic (LPO, GSH) markers in the cortex and hippocampus of healthy rats. Our results demonstrated that, with the single exception of CAT activity, the evaluated parameters (specifically LPO in the cortex and GPx activity in both brain structures) were significantly modulated only by the mRVT treatment. A similar trend and a more pronounced antioxidant capacity for the micellar formulation were also observed in our previous research under pathological conditions [26].

Despite the promising results regarding the activity of aqua micellar resveratrol, we acknowledge that the present study is limited by the fact that RVT and mRVT were administered in different vehicles—30% ethanol and an aqueous nanodispersion, respectively—due to solubility requirements. Consequently, the vehicle may have influenced the observed differences between the two treatments. Incorporating an ethanol-vehicle control arm in future research would help isolate and exclude this effect.

## 4. Materials and Methods

### 4.1. Preparation of Resveratrol-Loaded Micelles

The formation of resveratrol-loaded micelles was performed via the film hydration method. Particularly, 20 mg of Pluronic F127, 20 mg of Pluronic P123, and 5 mg trans-resveratrol were dissolved in 4 mL methanol. The solution was left at room temperature to fully evaporate the organic solvent. Thereafter, the resulting film was redispersed with distilled water, and the dispersion was filtered (0.2 µm Nylon filter). The amount of the non-encapsulated resveratrol was determined in the fraction obtained after rinsing the filter with 50% ethanol via UV-Vis spectrophotometric analysis at 306 nm (Thermo Fisher Scientific, Waltham, MA, USA). The mean diameter and PDI were evaluated by dynamic light scattering at a scattering angle of 90° (Zetasizer NanoBrook 90Plus PALS, Brookhaven Instruments Corporation, Holtsville, NY, USA). The zeta potential was measured by the phase analysis light scattering (PALS) method at a scattering angle of 15°.

### 4.2. Animals

Male Wistar rats (200–250 g) from Erboj Laboratories, Sofia, Bulgaria were used in this study in accordance with the requirements of the European Communities Council Directive (86/609/EEC) and the Bulgarian Food Safety Agency (approval for working with laboratory animals: permission No. 397/31.05.2029). The animals were housed under standard laboratory conditions (25 ± 3 °C, 12 h light/dark cycle) with full-time access to food and water. The experimental protocol started after a five-day habituation period.

### 4.3. Experimental Groups

Randomly selected rats were divided into 3 experimental groups (*n* = 9 for behavioral tests; *n* = 6 for biochemical analysis): control, RVT 10, and mRVT 10. A NaCl solution (0.9%, saline) was administered intraperitoneally (i.p.) as a vehicle to the control group. Resveratrol (Sigma Chemical Co., Schnelldorf, Germany) was dissolved in 30% ethanol and administered i.p. at a dose of 10 mg/kg to the RVT 10 group. Resveratrol-loaded micelles, as an aqueous nanodispersion, were administered i.p. at a dose of 10 mg/kg to the mRVT 10 group. The dose was selected based on positive results from previous publications [24,25,37,38]. All animals received daily injections for 9 consecutive days [36].

### 4.4. Behavioral Experiments

**Figure 8:**
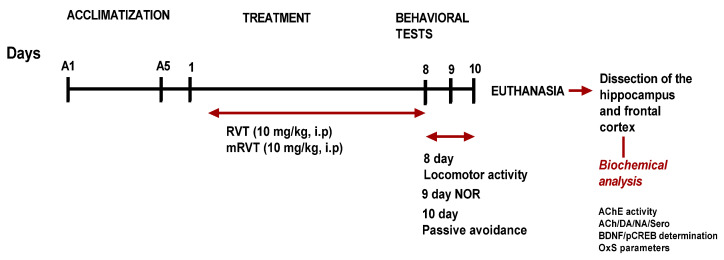
Experimental design.

The behavioral observations were made in a dimly lit room between 9 a.m. and 12 p.m. at the end of the experiment (Figure 8).

#### 4.4.1. Locomotor Activity Assessment

Locomotor activity was evaluated using a square open-field arena (60 cm × 60 cm × 60 cm), divided into nine equal sectors. Each rat was placed in the center of the arena, and its behavior was recorded for 3 min. Horizontal activity was quantified as the number of line crossings, while vertical activity was assessed by the number of rearings [39]. To ensure consistency, the apparatus was cleaned with 70% ethanol between test sessions to eliminate olfactory cues.

#### 4.4.2. Novel Object Recognition (NOR) Test

The novel object recognition (NOR) task is a widely accepted paradigm for evaluating short-term recognition memory deficits [40,41]. This test exploits the innate propensity of rodents to explore novel objects more than familiar ones and is independent of spatial reference memory [42]. The NOR test was conducted in an open-field arena (60 cm × 60 cm × 60 cm) over two consecutive days. On the first day (habituation phase), animals were allowed to freely explore the empty arena for 3 min, followed by a 3 min session with two identical objects placed at equal distances from the walls. On the second day (testing phase), rats explored the arena in the presence of two identical objects for 4 min. After a 1 h inter-trial interval, one of the familiar objects was replaced with a novel one, and the time that the animals spent exploring the familiar (F) and the novel (N) objects was recorded over a 3 min period. Recognition memory was quantified using a discrimination index (DI), which was calculated as DI = (N/(N + F)) × 100, where N and F represent the time that animals spent exploring novel and familiar objects, respectively [42].

#### 4.4.3. Passive Avoidance Test

This test is used for evaluation of short-term memory deficits of experimental animals via latency time of the reaction [43]. We used an automatic device (Ugo Basile, Gemonio, Italy), consisting of a two-compartment design with light-illuminated and darkened chambers, separated by a sliding door. The dark chamber is equipped with a stainless-steel grid floor. The test consists of training and retention sessions in the paradigm of short-term memory. 

During the acquisition (training) phase, each rat was placed in the light compartment and, following a 13 s door delay, they gained access to the dark compartment. When entering in the dark compartment (with all four paws), the door closed, and the rat was subjected to an electric foot-shock (0.7 mA for 3 s). The time taken to enter the dark chamber during this initial exposure was recorded as the initial latency (IL).

Retention sessions were conducted one hour post-training to evaluate short-term memory. During these trials, the same procedure was followed but the foot-shock was omitted. The step-through latency (STL)—the time elapsed before re-entering the dark compartment—was monitored, with a maximum cut-off time of 180 s assigned to animals that refrained from entering. These behavioral evaluations were performed on the 10th day of the experimental treatment.

### 4.5. Biochemical Analysis

#### 4.5.1. Tissue Preparation

One hour after the completion of behavioral testing, the animals were euthanized via mild CO_2_ inhalation and decapitated. Brains were quickly extracted on ice, and then the cortex and hippocampus were microdissected and briefly stored at − 25 °C prior to processing to minimize degradation of labile analytes, such as ACh, monoamines, and MDA. For biochemical assays, the isolated structures were minced into small pieces and rinsed in ice-cold phosphate-buffered saline (PBS, 0.01 mol/L, pH 7.4) to thoroughly remove excess blood. Tissue pieces were weighed and then homogenized in PBS (tissue weight g: volume PBS ml = 1:9) with ultrasonic homogenizer on ice. A cocktail of protease and phosphatase inhibitors (containing EDTA, EGTA, PMSF, NaF, Pepstatin A) was added to the buffer before homogenization, which aimed to slow down the proteolysis, dephosphorylation, and denaturation. All procedures were strictly conducted under temperature-controlled conditions (0 °C to +4 °C) to prevent protein degradation. Two distinct centrifugation protocols were employed to obtain the necessary fractions: For the low-speed fraction, a portion of the homogenate was centrifuged at 5000× *g* for 5 min. The resulting supernatant was utilized for the determination of acetylcholinesterase (AChE) activity, lipid peroxidation (LPO) levels, and glutathione (GSH) content, as well as for the quantification of BDNF, DA, NA, Sero, and pCREB levels. For the post-mitochondrial fraction, another portion was centrifuged at 12,000× *g* for 20 min. The obtained post-mitochondrial supernatant was collected for the assessment of antioxidant enzyme activities, including superoxide dismutase (SOD), catalase (CAT), and glutathione peroxidase (GPx).

#### 4.5.2. AChE Activity

AChE activity was assessed in the brains’ structures based on Ellman’s method [44].

#### 4.5.3. Neurotransmitter (ACh, DA, NA, Sero) and Signal Protein (BDNF, pCREB) Content

The brain levels of neurotransmitters and signaling proteins were quantified using commercially available enzyme-linked immunosorbent assay (ELISA) kits (pCREB (cat. no. SL1344Ra) was from Sunlong Biotech Co., Ltd. (Hangzhou, China). All other kits were from Elabscience (Wuhan, China)). The following markers were determined according to the manufacturers’ instructions, with acetylcholine (ACh) (cat. no. E-EL-0081), dopamine (DA) (cat. no. E-EL-0046), noradrenaline (NA) (cat. no. E-EL-0047), serotonin (Sero) (cat. no. E-EL-0033), BDNF (cat. no. E-EL-R1235) all from Elabscience (Wuhan, China), and pCREB (cat. no. SL1344Ra) from Sunlong Biotech Co., Ltd. (Hangzhou, China).

The optical density (OD) was measured at 450 nm using a microplate reader. The final results were calculated based on the corresponding standard curves and expressed in picograms per milliliter (pg/mL).

#### 4.5.4. Oxidative Stress Parameters

Oxidative stress biomarkers were quantified using commercially available assay kits from Sigma-Aldrich Co. LLC (Saint Louis, MO, USA), including the following: Lipid Peroxidation (MDA) Assay Kit (MAK085), Glutathione Assay Kit (CS0260), Superoxide Dismutase Determination Kit (19160), Catalase Assay Kit (CAT100), Glutathione Peroxidase Cellular Activity Assay Kit (CGP1).

#### 4.5.5. Protein Concentration

Protein concentrations in post-nuclear and post-mitochondrial tissue fractions were determined using the Lowry method [45], with bovine serum albumin used as the standard.

### 4.6. Statistical Analysis

All data were expressed as mean values ± SEM. The normality of distribution was confirmed using the Shapiro–Wilk and Kolmogorov–Smirnov tests. Differences among multiple experimental groups were analyzed by one-way analysis of variance (ANOVA), followed by Tukey’s post hoc test for multiple comparisons. Statistical significance was set at *p* < 0.05. The percentage differences between RVT and mRVT groups were calculated as: [(mRVT − RVT)/RVT] × 100. The effect size was calculated as eta-squared (η^2^ = SS_between/SS_total). Given the exploratory nature of the present study and the large number of outcome measures assessed across two brain regions, no correction for multiple comparisons was applied. Accordingly, the results should be interpreted as hypothesis-generating rather than confirmatory, and replication in independent cohorts is recommended before definitive conclusions can be drawn.

## 5. Conclusions

In conclusion, the present results demonstrate that micellar resveratrol exhibits a superior neurochemical profile compared to pure RVT across multiple systems in healthy rats. Specifically, mRVT produced more pronounced modulation of cholinergic neurotransmission, with higher elevation of ACh levels in the hippocampus. In the monoaminergic system, mRVT elicited significantly stronger increases in noradrenaline and serotonin levels in both the cortex and hippocampus, suggesting that enhanced monoaminergic support may be associated with micellar encapsulation. Furthermore, mRVT demonstrated a comparable upregulation with pure RVT of the BDNF/pCREB signaling axis in the cortex—key mediators of synaptic plasticity and neuronal resilience. Regarding oxidative stress, mRVT reduced cortical lipid peroxidation that was not observed with pure RVT. Critically, these broad neurochemical benefits were achieved without any adverse impact on locomotor activity or short-term memory, confirming a favorable safety profile of micellar formulation. Taken together, the present findings demonstrate that micellar encapsulation of resveratrol enhances its neurochemical efficacy under physiological conditions, most probably through improved CNS bioavailability, supported by the physicochemical characteristics of the formulation, particularly its small particle size (~33 nm) and better solubility. However, since neither plasma nor brain tissue concentrations of resveratrol were directly measured, this assumption remains unconfirmed.

## Figures and Tables

**Table 1:** Object exploration times in the NOR tests.

Group	Familiar Object Time (s)	Novel Object Time (s)
Control	1 ± 0 (*n* = 2)	11 ± 2.58 (*n* = 9)
RVT 10	6.67 ± 1.67 (*n* = 3)	10 ± 1.18 (*n* = 7)
mRVT 10	4 ± 0.95 (*n* = 5)	24.57 ± 3.88 (*n* = 7)

Values are presented as mean ± SEM, calculated only from animals with measurable (non-zero) exploration of the respective object.

## Data Availability

All data are available in the manuscript.

## Abbreviations

The following abbreviations are used in this manuscript:

RVT	Resveratrol (3,4′,5-trihydroxystilbene)
mRVT	Micellar resveratrol (resveratrol encapsulated in mixed Pluronic F127/P123 micelles)
ACh	Acetylcholine
AChE	Acetylcholinesterase
DA	Dopamine
NA	Noradrenaline
Sero	Serotonin
ROS	Reactive oxygen species
BDNF	Brain-derived neurotrophic factor
pCREB	Phosphorylated cAMP response element-binding protein
NOR	Novel object recognition
ELISA	Enzyme-linked immunosorbent assay
LPO	Lipid peroxidation
MDA	Malondialdehyde
GSH	Glutathione
SOD	Superoxide dismutase
CAT	Catalase
GPx	Glutathione peroxidase
PMSF	Phenylmethylsulfonyl fluoride
EDTA	Ethylenediaminetetraacetic acid
EGTA	Ethylenglycoltetaacetic acid
